# Elevated Fecal Calprotectin Accompanied by Intestinal Neutrophil Infiltration and Goblet Cell Hyperplasia in a Murine Model of Multiple Sclerosis

**DOI:** 10.3390/ijms242015367

**Published:** 2023-10-19

**Authors:** Mehrnaz Nouri, Björn Weström, Shahram Lavasani

**Affiliations:** 1 ImmuneBiotech AB, Medicon Village, 223 63 Lund, Sweden; 2 Department of Biology, Lund University, 223 62 Lund, Sweden; 3 Department of Clinical Sciences Lund, Lund University, 221 85 Lund, Sweden

**Keywords:** EAE, calprotectin, neutrophil, goblet cell, intestinal inflammation, IBS

## Abstract

Multiple sclerosis (MS) is an inflammatory demyelinating disease of the central nervous system caused by myelin-specific autoreactive T cells. We previously demonstrated intestinal barrier disruption and signs of inflammation in experimental autoimmune encephalomyelitis (EAE), a model of MS. Fecal calprotectin is a disease activity biomarker in inflammatory bowel diseases, released by neutrophils in response to inflammation. We aimed to further investigate EAE manifestations in the gastrointestinal tract and to determine whether calprotectin is a useful biomarker of intestinal inflammation in EAE. Calprotectin was analyzed in feces, cecal contents, and plasma of EAE mice. Infiltrating neutrophils and goblet cells were investigated in different parts of the gastrointestinal tract before the onset of neurological symptoms and during established disease. We found increased calprotectin levels in feces, cecal content, and plasma preceding EAE onset that further escalated during disease progression. Increased neutrophil infiltration in the intestinal tissue concomitant with IL-17 expression and myeloperoxidase activity was found to correlate well with clinical activity. Increased goblet cells in the intestine, similar to irritable bowel syndrome (IBS), were also observed. The results suggest calprotectin as a good biomarker of gastrointestinal inflammation in EAE and the potential of this model as a useful animal model for IBS.

## 1. Introduction

Multiple sclerosis (MS) is one of the most common immune-mediated demyelinating and neurodegenerative diseases of the CNS. However, the exact etiology is unknown. Functional gastrointestinal disorders in MS are common, and the prevalence is higher with longer disease duration [1]. There are also reports on increased incidence of inflammatory bowel diseases (IBD), including both Crohn’s disease (CD) and ulcerative colitis (UC), among MS patients, which indicates a role of the gastrointestinal (GI) tract in the development of the disease [2,3]. We have previously demonstrated increased permeability and inflammation of the intestine in mice with experimental autoimmune encephalomyelitis (EAE), an animal model of MS [4]. This finding was later confirmed by a clinical study on MS patients [5]. We also observed activation of Th17 cells and increased levels of other IL-17-producing cells in the lamina propria (LP) and the gut lymphoid organs [4].

Increasing amounts of evidence suggest a co-localization and cross-talk between T cells and neutrophils during inflammation [6,7]. Along with their beneficial role in innate immunity, recruitment and accumulation of neutrophils in the intestine is often associated with mucosal damage and disease development [8], suggesting that granulocytes may also play a role in intestinal pathology in MS. There is evidence suggesting a pathogenic role of neutrophils in development of extra-intestinal autoimmune diseases, e.g., in an MS model [9]. Cytokines released by Th17 cells in association with goblet cell alterations, as well as elevated neutrophil infiltration in the gut, followed by increased activity of myeloperoxidase (MPO), have been reported in IBD [10,11]. Elevated levels of IL-17 in the CSF and blood of MS patients correlate with clinical exacerbations and neutrophil infiltration into the CNS [12]. Neutrophils are essential in phagocytizing bacteria and the release of antimicrobial proteins, such as calprotectin, in the innate immune response. Calprotectin is a calcium- and zinc-binding heterocomplex of the S100A8 and S100A9 proteins, constituting approximately 60% of cytoplasmic content in neutrophils. Released calprotectin binds specifically to endothelial cells, leading to loss of barrier function [13,14]. Increased serum levels of calprotectin have previously been reported in several inflammatory conditions, including IBD, cystic fibrosis, rheumatoid arthritis, and multiple sclerosis [14,15,16,17,18]. As a consequence of intestinal inflammation, an increased concentration of calprotectin in feces is considered a marker of inflammation, which is used for the diagnosis and monitoring of IBD [19]. A mild increase in fecal calprotectin was also shown in different types of irritable bowel syndrome (IBS) [20,21]. Increased levels of calprotectin have also been detected in CSF of MS patients during the acute phase of the disease [22]. 

Based on these premises, we investigated the infiltration and activity of neutrophils in the intestine, examined the level of fecal calprotectin, and determined goblet cell numbers in EAE mice. We explored if fecal calprotectin might serve as a marker of intestinal inflammation in EAE as it does in gastrointestinal inflammatory disorders.

## 2. Results

### 2.1. Increased Calprotectin in Plasma, Cecal Content, and Feces 

**Figure 1:**
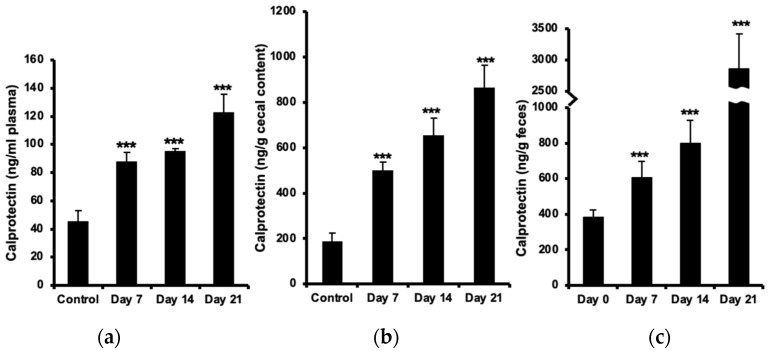
Increased calprotectin in blood plasma, cecal content, and feces during progression of EAE. Calprotectin was measured using an ELISA kit for levels in plasma (**a**), cecal content (**b**), and feces (**c**) in unimmunized animals (control) and in EAE mice before (day 0) or at 7 (day 7), 14 (day 14), and 21 days (day 21) after immunization. The results are expressed as mean ± SD (*n* = 7–10). *** indicates a statistical sign at *p*-value ≤ 0.001.

The concentration of plasma calprotectin increased (almost two-fold) already before the onset of neurological symptoms at day 7 post-immunization and further (up to three-fold) at day 21, as compared with healthy controls (Figure 1a). Calprotectin levels in cecal content were two-fold elevated at day 7 and more than four-fold at day 21 (Figure 1b). The increase in fecal calprotectin was even more marked, reaching an eight-fold increase (almost 3 µg/g feces) at day 21 (Figure 1c).

### 2.2. Increased Number of Neutrophils in the Intestine

**Figure 2:**
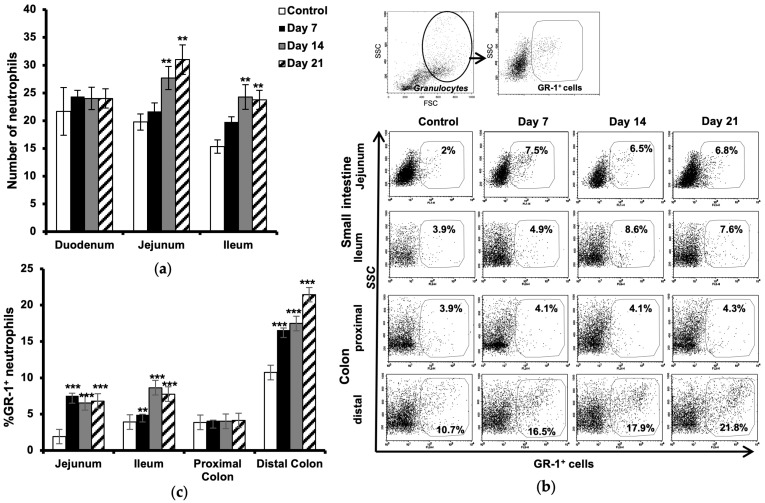
Increased number of neutrophils in the intestine during progression of EAE. Sections from the duodenum, jejunum, and ileum part of the small intestine, taken from unimmunized control animals and EAE mice at days 7, 14, and 21 post-immunization, were stained with H&E. Microscopical quantitative analysis of neutrophils in the lamina propria were performed (**a**). Each bar represents mean ± SD of neutrophil count in five randomly chosen villi from three different sections per animal (*n* = 4). Single-cell suspension from the jejunum, ileum, proximal, and distal colon at 7, 14, and 21 days after EAE induction were used for neutrophil analysis by flow cytometry. Gating was conducted sequentially for granulocytes (by light scattering properties) and the neutrophil surface marker GR-1 (**b**). Dot plots from one representative experiment from three experiments. Histogram shows relative percentage of Gr-1^+^ neutrophils in total gated granulocytes (**c**). The results are expressed as mean ± SD (*n* = 3). *** represents a *p*-value ≤ 0.001 and ** a *p*-value ≤ 0.01 in comparison with the controls.

Histological evaluation of neutrophils in the small intestine showed an increased number of these cells in lamina propria of the jejunum and ileum at the onset of the disease (day 7), which further augmented during the development of neurological symptoms (days 14 and 21 post-immunization) as compared to the control (Figure 2a).

Flow cytometric analysis of GR-1-expressing granulocytes confirmed a significant increase in neutrophils in the jejunum (more than three times) already at day 7 and with almost the same ratio during the progression of the disease. Increased numbers of neutrophils were observed in the ileum at day 7, which then increased more than two times at day 21. Further analysis in the colon revealed no significant changes in the number of neutrophils in the proximal part but showed an increased amount of these cells in the distal part at day 7, post-immunization, which then increased two folds at day 21 during the established and chronic phase of the disease (Figure 2b,c). All values are compared to results from unimmunized controls.

### 2.3. Increased IL-17 Expression in Intestinal Neutrophils

**Figure 3:**
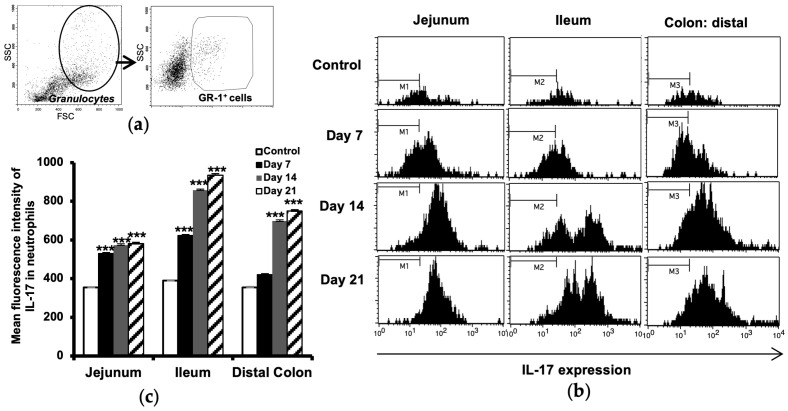
Increased IL-17 expression in intestinal neutrophils during progression of EAE. Single-cell suspensions from the small intestine (jejunum, ileum) and colon (distal) were used for flow cytometry analysis. The cells were gated as indicated for granulocyte (by light scattering properties) and further for expression of neutrophil surface marker GR-1 (**a**). The expression of IL-17 was then investigated in GR-1+ cells in the jejunum (**b**), ileum of the small intestine, and in the colon distal, taken from unimmunized controls and EAE mice at days 7, 14, and 21 after immunization. M1, 2, and 3 bars indicate the highest levels of IL-17 expression in cells isolated from control animals, representing a way to facilitate comparison between the groups. Data are representative of one of three independent experiments. Histogram shows mean fluorescence intensity of IL-17 in neutrophils (**c**). The results are expressed as mean ± SD (*n* = 3). *** represents a *p*-value ≤ 0.001 in comparison with the controls.

The IL-17 expression in neutrophils isolated from small intestinal and colon samples was analyzed using flow cytometry. The cell populations were sequentially gated for granulocytes (by light scattering properties), the neutrophil surface marker GR-1 (Figure 3a), and then for the expression of IL-17 (Figure 3b). The results showed a minor upregulation of IL-17 in neutrophils already before the onset of disease (day 7) in the jejunum and ileum and also in the distal colon, to become marked at days 14 and 21 (Figure 3c). No differences between EAE and control animals were observed in the proximal colon.

### 2.4. Enhanced Myeloperoxidase Activity in the Intestine

**Figure 4:**
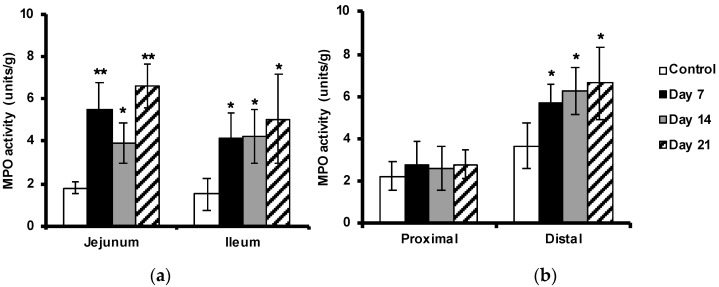
Increased MPO activity in the intestine during progress of EAE. MPO activity was measured in the jejunum and ileum parts of the small intestine (**a**) and the proximal and distal parts of the colon (**b**) in control unimmunized mice and EAE mice at days 7, 14, and 21 after immunization. A colorimetric method was used, and the results are expressed as mean ± SD (*n* = 4). * represents a *p*-value ≤ 0.05 and ** a *p*-value ≤ 0.01.

The MPO activity in the tissues was used to indicate neutrophil activation. MPO activity was increased in the small intestine and in the distal colon, but not significantly in the proximal colon, as compared with the non-immunized controls (Figure 4a,b). In concert with the previously observed neutrophil infiltration, MPO activity was already raised before the onset of the disease (at day 7) with more than two-fold in the small intestine and about 50% in the distal part of the colon, compared to samples isolated from control animals.

### 2.5. Increased Number of Intestinal Goblet Cells

**Figure 5:**
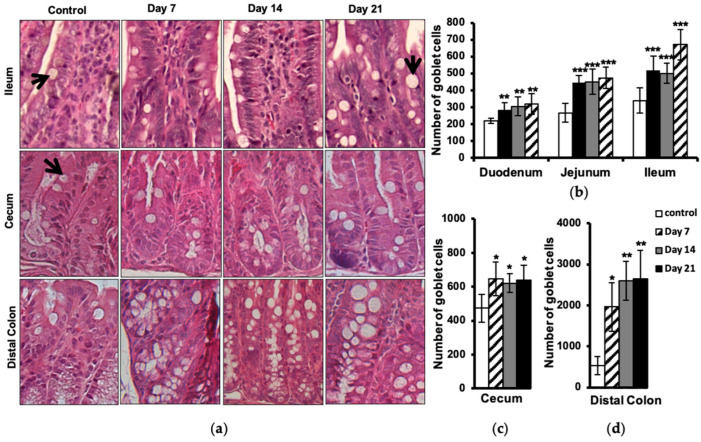
Increased goblet cell number in the intestine of EAE animals. H&E-stained sections from the ileum part of the small intestine cecum and distal colon (**a**) of unimmunized control and of EAE animals at days 7, 14, and 21 post-immunization (objective 20×), arrows show goblet cells. The amount of goblet cells was counted by performing microscopic quantitative analysis on sections from the duodenum, jejunum, and ileum of the small intestine (**b**), cecum (**c**), and distal colon (**d**). Each bar represents mean ± SD of three analyzed sections per animal (*n* = 4). * represents a *p*-value ≤ 0.05, ** a *p*-value ≤ 0.01, and *** a *p*-value ≤ 0.001.

Histological evaluation of goblet cells in different parts of the intestine showed an increased number of these cells in EAE animals at day 7 post-immunization, with some further elevation to be seen at day 21 in the small intestine (Figure 5a,b), cecum (Figure 5a,c), and distal colon (Figure 5a,d). No differences between EAE and control animals were observed in the proximal colon. 

## 3. Discussion

Fecal calprotectin is elevated in infectious and inflammatory conditions and has been suggested as a reliable, non-invasive marker that is specifically able to distinguish inflammatory from non-inflammatory conditions in different intestinal complications [23,24,25]. In this work, we report an increased level of fecal calprotectin in EAE mice, correlating temporally with disease development and innate immune alterations in the intestine. Our results correlate well with previous observations in CD and UC patients, indicating a strong association between fecal calprotectin levels and disease activities [26]. Furthermore, we also revealed increased levels of calprotectin in the cecum contents and blood plasma of these animals. The data showed changes in calprotectin concentrations early during the disease development, which then increased during the progress of the disease. The results are comparable with previous observations in MS patients showing elevated levels of calprotectin in plasma and CSF, which correlated well with the disease activity and the presence of phagocyting cells in the active CNS lesions [22,27,28].

Since increased intestinal permeability has been documented in patients with IBD, and luminal calprotectin excretion reflects the migration of neutrophils through the intestinal epithelium via intercellular junctions, a relationship between intestinal permeability and fecal calprotectin has been suggested [29]. We have previously reported increased intestinal permeability and an altered small intestinal mucosal structure, including inflammation in the EAE mice [4]. Increased intestinal permeability in patients with multiple sclerosis has also been shown previously, but no significant increase in calprotectin, which could be due to the anti-inflammatory medication [5,30]. In fact, it has been shown that Natalizumab, a common medication to decrease relapse frequencies in relapsing-remitting MS, can reduce intestinal inflammation with no effect on intestinal permeability [31].

In order to explore a correlation between increased intestinal permeability and luminal calprotectin excretion, we further examined the GI tract and found increased numbers of infiltrating neutrophils in the jejunal and ileal part of the small intestine and the distal part of the colon. Increased MPO activity in these intestinal segments confirmed the accumulation and activation of neutrophils in EAE mice. MPO activity has been shown to be directly relative to the neutrophil number in animal models of intestinal inflammation [32]. MPO is the most abundant peroxidase enzyme in the neutrophil azurophilic granules, which is synthesized during myeloid differentiation and, upon activation, is involved in the induction of neutrophil apoptosis [33,34]. Previous results indicate that the proinflammatory cytokines, e.g., IFN-γ and TNF-α, or other stimuli may regulate mucosal barrier function and provide signals for the recruitment and activation of neutrophils and other inflammatory cells [4,29]. Epithelial damage may also reduce transepithelial resistance and allow leakage of luminal material into the lamina propria. A phenomenon called ‘leaky gut’ has been suggested as a preexisting primary mucosal defect in CD and has previously been demonstrated by us in EAE mice [4,35]. Our results show increased neutrophil recruitment to the intestinal tissue starting early during the progress of CNS inflammation, concomitant with increased IL-17 expression and MPO activity. There is evidence of cross-talk between Th17 cells and activated neutrophils where they can trigger activation and reciprocal recruitment via the release of chemokines [7]. Th17 cells were found in gut tissue from CD and synovial fluid from rheumatoid arthritis patients [7,36]. Recent studies have also demonstrated the key role of these cells in the development of EAE during the early stages of the disease [9]. Activated neutrophils are accumulating in the CNS, both prior to and during the acute phase of EAE. IL-17 has been suggested to trigger pathogenic pathways mediating the disruption of the blood–brain barrier and release of neutrophil-recruiting cytokines and growth factors leading to the accumulation of neutrophils in the CNS. We have previously shown increased amounts of IL-17-producing T lymphocytes, including both Th17 and γδ T cells, in the gut and associated lymphoid tissues in the EAE mice. Interestingly, our findings also revealed increased levels of IL-17-expressing CD3^−^ lymphocytes, which we hypothesized to be involved in EAE pathology [4]. Type 3 innate lymphoid cells (ILC3) have been identified as another important source of IL-17 in the intestine. These cells are also involved in neutrophil recruitment to the site of inflammation [37]. This may explain our observation of increased infiltration of neutrophils into the GI tract of these animals. Our further analysis of these neutrophils also revealed a markedly upregulated expression of IL-17, which may be triggered as an outcome of the available cytokine milieu and confirm the previous report on increased neutrophils in the colonic tissue in a spontaneous model of EAE [38]. There is also evidence indicating that IL-17 may stimulate the release of MPO from neutrophils [39], which further explains the increased MPO activity we observed in the Intestinal tissues of EAE mice.

Goblet cells are present in the whole intestine and constitute the main source of mucins, providing a dynamic protecting barrier influenced by inflammatory conditions [40]. Goblet cell hyperplasia has been described in a number of infections caused by parasites, bacteria, and viruses. They play an important role in gut barrier defense, mainly by producing mucin, and they participate in immune responses by presenting luminal antigens to lymphoid cells. An important cytokine, IL-22, produced by Th17 cells, has been identified as a potent inducer of goblet cell mucus production [41,42]. Additionally, ILC3, representing an innate counterpart to Th17 cells, stands out as another important source of IL-22 in the intestine. ILC3s have the capacity to stimulate mucin production by goblet cells; however, it is noteworthy that their influence mainly depends upon the specific microenvironment [43]. Nevertheless, the depletion of goblet cells and their mucus production is a hallmark of IBD pathology [40]. It may be speculated that the goblet cell depletion seen in IBD is a late manifestation of a long-standing and chronic inflammatory condition.

We report the reverse situation in EAE, a significant increase in goblet cell number, correlating with accumulation of intestinal neutrophils. The goblet cell hyperplasia we observed in the gut of EAE mice may be mediated by increased activation of Th17 cells and release of their cytokines, in turn leading to recruitment of neutrophils and goblet cell hyperplasia. Furthermore, our finding may explain the increase in Akkermansia muciniphila in EAE and MS patients. Akkeramensia consume mucin, and with the increased amount of goblet cells, there is more food for this bacterium, which favors an increased amount of Akkeramensia. Increased goblet cells mostly indicate the presence of danger in the gut lumen due to, e.g., bacterial invasion and exposure to LPS, which triggers goblet cell hyperplasia and subsequent mucus production, flushing out the bacteria [44]. In our previous study, we showed an affected intestinal mucosal barrier with increased permeability early during the development of EAE. We have further demonstrated an altered tight junction regulation and hypothesized an increased paracellular permeability, exposing the body more to environmental factors and harmful microbial invasion, promoting intestinal inflammation [4]. This scenario may stimulate the proliferation and differentiation of intestinal goblet cells and also result in the recruitment of phagocytes, e.g., neutrophils, to actively implicate and begin the immunological defense against the invasion. Observations from IBD patients have shown increased neutrophil migration into the gut, leading to apoptosis and the release of calprotectin as a consequence of intestinal inflammation [29].

In conclusion, we have shown that intestinal components of innate immunity are affected in EAE, including neutrophils and goblet cells, in conjunction with elevated calprotectin concentrations. Our data prompt a study on fecal calprotectin as a clinically useful marker of intestinal inflammation and possibly also as a marker of neurological disease activity in MS patients. Furthermore, similarities between IBS and our findings in the EAE could suggest that EAE is a good model for IBS. However, further investigation is needed.

## 4. Materials and Methods

### 4.1. Animals

Female C57BL/6 mice (8–10 weeks old) were obtained from (Taconic M & B A/S, Ejby, Denmark). The mice were bred under specific pathogen-free conditions in a controlled environment (20 ± 1 °C, 50 ± 10% relative humidity, 12:12 h light–dark cycle). The trials followed the European Community regulations for animal experiments and were approved by the local Ethical Review Committee for Animal Experiments (Permit Number: M211-11).

### 4.2. Induction and Assessment of Experimental Autoimmune Encephalomyelitis (EAE)

A synthetic myelin peptide oligodendrocyte glycoprotein (MOG), amino acids 35–55 (MEVGWYRSPFSRVVHLYRNGK-COOH, Schafer-N, Copenhagen, Denmark) was used to induce EAE. Mice were immunized by intradermal injection with an emulsion containing 100 µg of the peptide in complete Freund’s adjuvant (H37RA, Difco Laboratories, Franklin Lakes, NJ, USA) together with i.p. injections of 200 ng pertussis toxin (Sigma-Aldrich, Stockholm, Sweden) at days 0 and 2. To follow the progression of the disease, the animals were weighed and examined daily for clinical signs of EAE in a blinded fashion [45]. Since a disease incidence of approximately 80% is expected and the animals usually lose around 10% of their body weight, preceding by a few days the disease onset, which appears 8–10 days after immunization, only mice with weight loss were examined at day 7. By days 14 and 21, animals with signs of disease (1 ≤ score) were included in the experiment (Figure S1). Unimmunized healthy mice were used as controls. At the end of the experiments, the animals were anesthetized with a mixture of Ketamin (0.5 mg/g body weight; Ketalar, Pfizer AB, Stockholm, Sweden) and Azaperon (0.4 mg/g body weight; Stresnil, Janssen-Cilag Pharma, Wien, Austria), and blood was taken from the vena cava into tubes containing 1.5 mg EDTA and 20 000 IU aprotinin (Trasylol, Bayer, Leverkusen, Germany) that were ice-chilled until centrifugation at 3000× *g* for 15 min to separate the plasma. The small intestine, cecum, cecal content, and colon from each animal were dissected for analysis.

### 4.3. Calprotectin Measurement

Feces were collected by temporally moving the animal to a separate clean cage on day 0 (before inducing the disease) and days 7, 14, and 21 after inducing EAE. The samples were kept at −20 °C until analysis. The concentration of calprotectin was determined by using a commercially available S100A8/S100A9 enzyme-linked immunosorbent assay (ELISA) kit (Immundiagnostik, Bensheim, Germany). A 4-parameter algorithm was used to form the standard curve and to calculate data. Data were presented in ng/mL for plasma and in ng/g for feces and cecum content.

### 4.4. Microscopic Analysis of Neutrophils and Goblet Cells

Dissected small intestine, cecum, and colon from 4 animals in each group (Control, EAE Day 7, Day 14, Day 21) were washed with phosphate-buffered saline (PBS) and fixed in 4% phosphate-buffered formaldehyde for 24 h and then stored in 70% ethanol until further processing. After embedding into paraffin, the tissues were cut laterally into 5 µm thick sections, deparaffinized, and stained with hematoxylin and eosin (H&E) according to standard procedures. The sections were photographed by using an Olympus PROVIS microscope (objective 20× for goblet cells and 40× for neutrophils) equipped with an Olympus DP50 camera (Olympus, Tokyo, Japan). Cells were counted by using the ImageJ software v1.51w (NIH, Bethesda, MD, USA). The number of goblet cells was determined (counting vacuoles) in three entire sections from the duodenum (the most proximal), jejunum (middle part), and ileum (the most distal) part of the small intestine, cecum as well as the proximal and distal part of the large intestine from each animal. Neutrophils were counted in the lamina propria of 5 randomly chosen villi from 3 different sections in each part of the small intestine from the animals at either 7, 14, or 21 days after EAE induction and from the healthy control animals.

### 4.5. Flow Cytometry Analysis of Neutrophils

Flow cytometry analysis of neutrophils in the small intestine and colon was performed using our previous protocol [42]. In brief, at the end of each experiment, the mouse intestine was isolated, cleaned from fat and connective tissues, washed thoroughly with PBS to remove all content, opened longitudinally, cut into 0.5 cm pieces (the parts with Peyer’s patches were excluded) and shaken at 220 rpm in 25 mL EDTA solution for 30 min at 37 °C. Samples were centrifuged at 1500× *g*, and the supernatant was discarded; this process was repeated twice. The tissues were then washed with harvest medium consisting of RPMI 1640, heat-inactivated fetal bovine serum, HGPG (HEPES, L-glutamine, penicillin/streptomycin, and gentamycin) for 5 min before incubation with collagenase (100 U/mL) for 45 min at 37 °C on the shaker followed by centrifugation. All cells were then incubated with anti-CD16/CD32 followed by FITC-conjugated anti-mouse Ly-6G (Gr-1) for neutrophils. For analysis of intracellular cytokines, cells were fixed with Cytofix/Cytoperm solution and stained with PE-conjugated anti-IL-17A (eBioscience, San Diego, CA, USA). A FACSort flow cytometer was used for the acquisition of data, and analysis was made using CELLQuest software v5.2.1 (BD Biosciences, San Diego, CA, USA) [42].

### 4.6. Myeloperoxidase Activity

Tissue sections from the proximal and distal parts of the small and large intestines were collected after being flushed with PBS to clean from their contents and snap-frozen in isopentane on dry ice and then stored at −80 °C until measurements. Samples were weighed, homogenized in 0.02 M phosphate buffer (PB), pH 7.4, and centrifuged at 13,500× *g* for 5 min. The pellet was resuspended in 0.05 M PB, pH 6.0, containing 0.5% hexadecyltrimethylammonium bromide and frozen at −20 °C for 2 h, then thawed at 25 °C, followed by sonication. Thereafter, the samples were placed in a water bath at 60 °C for 2 h and then centrifuged again. The supernatants were used, and a tetramethylbenzidine liquid substrate system (Sigma-Aldrich) was added as a peroxidase substrate, and the mixture was incubated in the dark for 10 min at room temperature. To stop the reaction, 0.5 M H_2_SO_4_ was used. The enzyme activity was determined spectrophotometrically, as the MPO catalyzed change in absorbance at 450 nm in the redox reaction of H_2_O_2_ at 25 °C. Values are expressed as MPO units per gram of tissue.

### 4.7. Statistics

Statistical evaluation was performed using StatView software v5.0 (SAS, Campus Drive Cary, NC, USA). Calprotectin and MPO activity data were analyzed by using ANOVA with Bonferroni/Dunn testing. The results of the histological analysis were compared using a nonparametric Mann–Whitney test. In all statistical analyses, *p* ≤ 0.05 was taken as the level of significance.

## Data Availability

The data are available on request from the corresponding author.

## Supplementary Materials

The following supporting information can be downloaded at https://www.mdpi.com/article/10.3390/ijms242015367/s1.
