# Supplementary material for: Elevated Fecal Calprotectin Accompanied by Intestinal Neutrophil Infiltration and Goblet Cell Hyperplasia in a Murine Model of Multiple Sclerosis
Source: Int J Mol Sci. 2023 Oct 19;24(20):15367. doi: 10.3390/ijms242015367 (PMC10606994; doi:10.3390/ijms242015367)
Supplement: Supplementary file 1 [file ijms-24-15367-s001.zip › ijms-2624664-supplementary.pdf]

Figure S1:

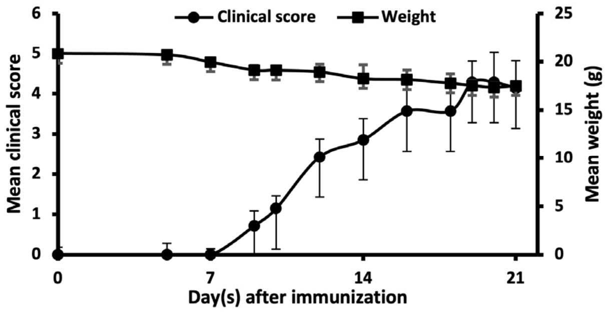

(**a**)

Disease scoring in EAE model used in this work:

0 : No symptoms
1 : Weak tail
2 : Paralyzed tail
3 : Paresis and gait disturbance
4 : Paralysis of one limb
5 : Paralysis of two limbs
6 : Two limbs paralyzed + paresis in a third
7 : Three limbs paralyzed, quadriplegia

(**b**)

Figure S1. Disease progress in EAE mice used in this study. Animals usually lose around 10% of their body weight, preceding by a few days the disease onset, which appears 8–10 days after immunization, only mice with weigh loss were examined at day 7. By day 14 and 21, animals with signs of disease (1≤ score) were included in the experiment (a). Data represent the mean±SD clinal score and weight of EAE animals (n=10). Disease scoring strategy in EAE model used in this study (b).
